# Advances in the Diagnosis and Management of High-Risk Cardiovascular Conditions: Biomarkers, Intracoronary Imaging, Artificial Intelligence, and Novel Anticoagulants

**DOI:** 10.3390/jcdd13010052

**Published:** 2026-01-19

**Authors:** Clarissa Campo Dall’Orto, Rubens Pierry Ferreira Lopes, Gilvan Vilella Pinto, Pedro Gabriel Senger Braga, Marcos Raphael da Silva

**Affiliations:** 1 Department of Hemodynamics, Brazilian Society for Health Support Hospital, Teixeira de Freitas 45995-000, Brazildrgilvanvilella@gmail.com (G.V.P.F.); marrabahia@hotmail.com (M.R.d.S.); 2 Department of Hemodynamics, AMES Hospital, Eunápolis 45820-131, Brazil; 3 Department of Hemodynamics, Costa das Baleias State Hospital, Teixeira de Freitas 45990-566, Brazil; 4 Pro-Coracao Clinic, Sao Paulo 05021-010, Brazil; pedro.senger@usp.br; 5 Cardio-Oncology Department, Heart Institute, University of São Paulo, São Paulo 05403-900, Brazil

**Keywords:** acute coronary syndrome, thrombosis, biomarkers, intracoronary imaging, optical coherence tomography, artificial intelligence, factor XI inhibitors, risk stratification

## Abstract

Understanding thrombosis in acute coronary syndromes (ACSs) has evolved through advances in biomarkers, intracoronary imaging, and emerging analytical tools, improving diagnostic accuracy and risk stratification in high-risk patients. This narrative review provides an integrative overview of contemporary evidence from clinical trials, meta-analyses, and international guidelines addressing circulating biomarkers, intracoronary imaging modalities—including optical coherence tomography (OCT), intravascular ultrasound (IVUS), and near-infrared spectroscopy (NIRS)—artificial intelligence–based analytical approaches, and emerging antithrombotic therapies. High-sensitivity cardiac troponins and natriuretic peptides remain the most robust and guideline-supported biomarkers for diagnosis and prognostic assessment in ACS, whereas inflammatory markers and multimarker strategies offer incremental prognostic information but lack definitive validation for routine therapeutic guidance. Intracoronary imaging with IVUS or OCT is supported by current guidelines to guide percutaneous coronary intervention in selected patients with ACS and complex coronary lesions, leading to improved procedural optimization and clinical outcomes compared with angiography-guided strategies. Beyond procedural guidance, OCT enables detailed plaque characterization and mechanistic insights into ACS, while NIRS provides complementary information on lipid-rich plaque burden, primarily for risk stratification based on observational evidence. Artificial intelligence represents a rapidly evolving tool for integrating clinical, laboratory, and imaging data, with promising results in retrospective and observational studies; however, its clinical application in thrombosis management remains investigational due to the lack of outcome-driven randomized trials. In the therapeutic domain, factor XI inhibitors have demonstrated favorable safety profiles with reduced bleeding and preserved antithrombotic efficacy in phase II and early phase III studies, but their definitive role in ACS management awaits confirmation in large, outcome-driven randomized trials. Overall, the integration of biomarkers, intracoronary imaging, and emerging analytical and pharmacological strategies highlights the potential for more individualized cardiovascular care. Nevertheless, careful interpretation of existing evidence, rigorous validation, and alignment with guideline-directed practice remain essential before widespread clinical adoption.

## 1. Introduction

The diagnosis of thrombosis, particularly in acute coronary syndromes (ACS) and other high-risk cardiovascular settings, has evolved with the incorporation of biomarkers, advanced imaging techniques, and artificial intelligence. Biomarkers such as D-dimer, troponins, and proteomic panels have enabled more precise stratification of thrombotic risk and support therapeutic decision-making, although the clinical integration of novel biomarkers still faces challenges related to validation and implementation [1,2]. Imaging tools such as coronary computed tomography angiography, cardiac magnetic resonance imaging, and intracoronary optical coherence tomography (OCT) provide high sensitivity for thrombus detection and for assessing ischemic risk [3].

Artificial intelligence has been applied to image analysis and thrombotic risk prediction, with machine-learning models demonstrating superior performance compared with traditional clinical risk scores in heterogeneous populations, although external validation and integration into clinical workflows remain limited [4].

In antithrombotic therapy, important advances have been achieved with the use of potent P2Y12 inhibitors, aspirin de-escalation strategies, and the introduction of direct oral anticoagulants (DOACs), which offer an improved safety profile in patients with complex comorbidities such as renal insufficiency or cancer [3,5,6]. The European Society of Cardiology, for example, recommends reducing the duration of triple therapy in patients with atrial fibrillation following an acute coronary syndrome, prioritizing DOACs and P2Y12 inhibitors to minimize bleeding [3]. Individualized strategies, based on tools such as the DAPT score, genotyping, and platelet function testing, are increasingly used to balance ischemic and bleeding risks [7,8,9].

Future perspectives include the development of anticoagulants targeting pathways such as factor XI. Emerging pharmacological strategies, particularly factor XI inhibitors, aim to dissociate antithrombotic efficacy from bleeding risk, representing a promising option for high-risk patients or those with contraindications to traditional anticoagulants. These agents have the potential to reduce thrombotic events without increasing bleeding risk, and their use may be further enhanced by the integration of biomarkers and AI-based algorithms to personalize treatment [10,11]. The current trend points toward the convergence of molecular diagnostics (including advances in biomarkers such as plasma proteomics and multimarker panels), comprehensive thrombotic and bleeding risk stratification, and personalized pharmacological therapy, with the goal of maximizing efficacy and safety in patients with complex clinical profiles.

This manuscript is intentionally presented as a narrative, conceptually integrative review, rather than as an exhaustive systematic review. Our objective is to synthesize representative evidence, contemporary guidelines, and pathophysiologic concepts that collectively inform clinical decision-making in acute coronary syndromes (ACS) and other high-risk coronary scenarios. Within this framework, biomarkers, intracoronary imaging, emerging artificial intelligence–based analytics, and evolving antithrombotic strategies are not discussed as isolated domains, but rather as interconnected components of a unified decision pathway.

## 2. Methods

A targeted, non-systematic literature search was conducted to support the conceptual framework of this narrative review. The search was performed in PubMed/MEDLINE, Embase, and the Cochrane Library, prioritizing publications from January 2019 to March 2025, while allowing inclusion of seminal earlier studies when necessary to provide historical or mechanistic context. Search terms included combinations of “acute coronary syndrome,” “arterial thrombosis,” “high-sensitivity troponin,” “natriuretic peptides,” “intracoronary imaging,” “intravascular ultrasound,” “optical coherence tomography,” “near-infrared spectroscopy,” “artificial intelligence in cardiology,” and “factor XI inhibitors”.

Studies were selected based on clinical relevance, methodological rigor, and contribution to an integrative understanding of diagnosis, risk stratification, and therapeutic decision-making in acute coronary syndromes. Given the narrative and integrative nature of this review, formal systematic review methods and quantitative meta-analysis were not applied.

## 3. Biomarkers

It is important to note that, except for cardiac troponins for the diagnosis of acute myocardial infarction, most circulating biomarkers discussed in this section primarily provide prognostic and risk stratification information rather than direct therapeutic guidance. While several biomarkers and multimarket models have demonstrated incremental predictive value in observational studies, their routine use to guide specific treatment strategies is not uniformly supported by outcome-driven randomized trials or formal guideline recommendations.

The personalization of antithrombotic therapy in patients with acute coronary syndrome, high cardiovascular risk, and multiple comorbidities depends on the integration of biomarkers that enhance risk stratification beyond traditional clinical models [8,12,13,14]. Conventional models based on clinical characteristics and scores such as the dual antiplatelet therapy (DAPT) have limitations in capturing patient heterogeneity, particularly in high-risk settings and in the presence of complex comorbidities [8,14].

High-sensitivity cardiac troponin (hs-cTn) remains central to the diagnosis of acute myocardial infarction and provides robust prognostic information in ACS [15,16,17,18,19]. Measurement of hs-cTn is recommended for the diagnosis of acute myocardial infarction in patients with suspected ACS (Class I, Level of Evidence A) [15,16]. When contemporary high-sensitivity assays are used, diagnostic sensitivity exceeds 90–95% at presentation or within validated serial testing algorithms, with a negative predictive value above 99% for ruling out myocardial infarction; specificity is lower (approximately 80–90%) due to myocardial injury related to non-ischemic conditions [15]. Beyond diagnosis, higher absolute troponin concentrations and dynamic changes over time are consistently associated with an increased risk of recurrent ischemic events and all-cause mortality [18,19,20,21].

Natriuretic peptides, including B-type natriuretic peptide (BNP) and N-terminal pro–B-type natriuretic peptide (NT-proBNP), are not diagnostic markers of ACS but provide strong prognostic information. In patients with ACS, elevated BNP or NT-proBNP levels are independently associated with short- and long-term mortality, incident heart failure, and risk of rehospitalization, even after adjustment for troponin levels and left ventricular systolic function [17,18,19,20,21]. Their prognostic value is particularly relevant in patients with concomitant heart failure, diabetes, or renal dysfunction. Although no universal cutoff applies across all clinical scenarios, NT-proBNP values above age-adjusted thresholds (e.g., >300–600 pg/mL in acute settings) consistently identify patients at increased risk of adverse outcomes [18,19,22].

Homocysteine has been investigated as a marker of endothelial dysfunction and thrombotic risk. Plasma homocysteine concentrations above approximately 15 μmol/L are associated with an increased risk of arterial thrombosis and cardiovascular events, with reported odds ratios ranging from 1.3 to 1.6 in observational studies [17,23]. In the context of ACS, homocysteine may provide incremental prognostic information when combined with troponin or inflammatory biomarkers [21,22]; however, its sensitivity and specificity are modest, and routine measurement is not recommended outside selected research settings or high-risk populations [21,23,24]. Inflammatory biomarkers, including high-sensitivity C-reactive protein (hsCRP) and interleukin-6 (IL-6), reflect systemic inflammatory activation and plaque instability. Elevated hsCRP levels, typically >2–3 mg/L, are associated with an increased risk of recurrent ischemic events and mortality after ACS, although their diagnostic sensitivity and specificity are low, limiting their role to prognostic assessment [14,21,22,23,24]. IL-6 has shown stronger associations with cardiovascular mortality and recurrent events, but similarly lacks sufficient specificity to guide therapeutic decisions in routine clinical practice.

Growth differentiation factor-15 (GDF-15) is a stress-responsive cytokine that has emerged as a powerful prognostic biomarker in ACS. Elevated GDF-15 concentrations are consistently associated with increased all-cause mortality, bleeding risk, and composite ischemic outcomes, with prognostic performance largely independent of troponin and natriuretic peptides [25]. However, GDF-15 is a nonspecific marker of cellular stress and inflammation, and no validated cutoff currently exists to support therapeutic decision-making. The integration of multiple biomarkers into composite models or personalized risk algorithms enhances the precision of risk stratification and may support individualized clinical reasoning, particularly in patients with multiple comorbidities. Multimarker models combining troponin, natriuretic peptides, inflammatory markers, and platelet activation biomarkers have demonstrated improved discrimination for adverse events when applied early after presentation, although their incremental benefit over established clinical tools varies across studies [21,23]. Despite these advances, the routine implementation of most emerging biomarkers in international guidelines remains limited. With the exception of troponins and, to a lesser extent, natriuretic peptides, most biomarkers lack prospective validation in randomized clinical trials and have not been formally incorporated into guideline-directed therapeutic algorithms [8,19,24]. Emerging omics-based biomarkers and artificial intelligence–driven integration strategies hold promises for future personalization of antithrombotic therapy but currently remain within the translational research domain [14,24].

In summary, as shown in Table 1, high-sensitivity cardiac troponin and natriuretic peptides represent the biomarkers with the strongest clinical evidence for routine use in ACS, whereas homocysteine, inflammatory markers, GDF-15, sCD40L, and multimarker panels provide incremental prognostic information in selected high-risk patients but await definitive validation and formal incorporation into international guideline recommendations.

## 4. Intracoronary Imaging in ACS and High-Risk Patients: Advances in Integration with Artificial Intelligence

It is important to recognize that the strength of evidence and the level of guideline support vary substantially across intracoronary imaging modalities and clinical applications, as shown in Table 2. While intravascular imaging–guided percutaneous coronary intervention (PCI) is supported by contemporary guidelines in selected settings, other uses of intracoronary imaging, including plaque characterization and vulnerability assessment, are primarily supported by observational and mechanistic studies and should be interpreted within this evidentiary context.

### 4.1. Recent Evidence and Guidelines for Intracoronary Imaging in ACS and Complex Lesions

Contemporary guidelines from the American College of Cardiology, the American Heart Association, and the Society for Cardiovascular Angiography and Interventions support the use of intravascular ultrasound (IVUS) or optical coherence tomography (OCT) to guide stent implantation in selected patients with acute coronary syndrome (ACS) and complex coronary anatomy, including left main disease, bifurcation lesions, and long lesions [23,24]. In this setting, IVUS- or OCT-guided PCI is recommended to optimize stent expansion and apposition, minimize malposition, and reduce procedural complications such as edge dissection, thereby improving clinically meaningful outcomes when compared with angiography-guided PCI (Class IIa, Level of Evidence A) [16,26]. Importantly, this guideline support specifically applies to the use of IVUS and OCT as procedural guidance tools during PCI. Other applications discussed in this section, such as plaque composition analysis and vulnerability assessment, are not currently endorsed by guidelines as indications for outcome-driven therapeutic interventions.

### 4.2. Near-Infrared Spectroscopy (NIRS)

**Figure 1:**
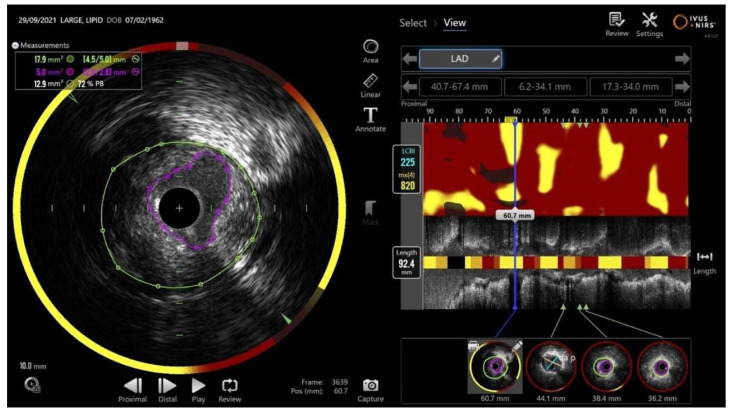
Images from the MAKOTO system (Nipro Medical Corporation): IVUS image (**left**) and NIRS image (**right**). On the (**right**), the chemogram analyzes lipid-rich plaques using a proprietary score ranging from 0 to 1000 and automatically highlights the most critical 4 mm of the lesion.

Near-infrared spectroscopy (NIRS), typically combined with IVUS, is useful for identifying vulnerable plaques with a high lipid content, which are associated with an increased risk of future events (Figure 1). Observational studies and multicenter registries have shown that lesions with a high lipid burden index (maxLCBI ≥ 400) identified by NIRS-IVUS are associated with a higher incidence of adverse cardiovascular events, including cardiac death, reinfarction, and revascularization, whereas non-lipidic plaques have a more favorable prognosis [27,28]. However, after PCI with contemporary drug-eluting stents, the presence of lipid-rich plaque detected by NIRS was not associated with an increased rate of periprocedural or late adverse events compared with non-lipidic plaques [29]. Accordingly, the prognostic value of NIRS appears to reside primarily in risk stratification and identification of patients with a higher likelihood of future events, rather than in guiding immediate procedural or therapeutic decisions [27,28,29,30].

To date, there are no large randomized clinical trials directly comparing NIRS with IVUS or OCT with respect to hard clinical endpoints such as mortality, reinfarction, or stent thrombosis. The current evidence base for NIRS is derived predominantly from observational studies and registries, and prospective validation in dedicated outcome-driven trials is still required [27,30]. In addition, many IVUS-, OCT-, and NIRS-based studies have been conducted in tertiary referral centers, with underrepresentation of women and ethnic minorities, which may limit the generalizability of the findings.

In summary, IVUS and OCT are supported by contemporary guidelines for guiding PCI in ACS and complex coronary lesions, with demonstrated reductions in ischemic events and repeat revascularization when compared with angiography-guided PCI [26]. In contrast, NIRS provides complementary information for risk stratification based on lipid burden, but its direct impact on clinical outcomes remains investigational and awaits confirmation in randomized comparative studies.

### 4.3. Optical Coherence Tomography (OCT)

Optical coherence tomography (OCT) is a high-resolution intracoronary imaging modality that allows detailed visualization of the coronary vessel wall, characterization of atherosclerotic plaque morphology, and identification of features such as thrombus, fibroatheroma, plaque rupture, neointimal tissue, and post–stent implantation complications, including malapposition and edge dissection. Compared with coronary angiography, which provides a two-dimensional assessment of the lumen, and with intravascular ultrasound (IVUS), OCT offers superior spatial resolution, enabling more precise assessment of superficial plaque characteristics, calcium thickness, lipid content, and intracoronary thrombus. These properties make OCT particularly useful for mechanistic evaluation in acute coronary syndromes and in complex coronary lesions [16,31,32].

The main limitations of OCT include its lower tissue penetration compared with IVUS, the requirement for contrast injection to achieve adequate blood clearance, and reduced applicability in certain coronary segments, such as ostial lesions or in patients with significant renal dysfunction [26,30].

Randomized clinical trials and contemporary studies, including RENOVATE-COMPLEX-PCI, OCTOBER, and OCCUPI, have demonstrated that OCT-guided stent implantation in selected patients with complex coronary lesions is associated with a reduction in adverse clinical events, including cardiac death, myocardial infarction, stent thrombosis, and repeat revascularization, when compared with angiography-guided PCI [33,34]. Accordingly, the use of OCT to guide PCI in patients with ACS and complex coronary anatomy is supported by contemporary guidelines as a reasonable strategy to optimize procedural outcomes [26].

**Figure 2:**
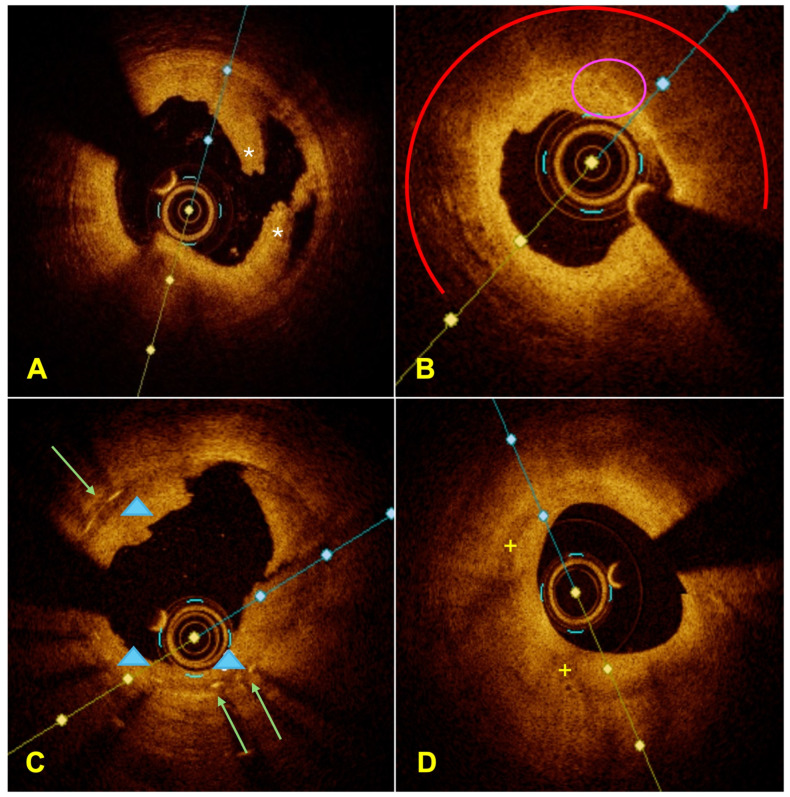
(**A**) OCT image of plaque dissection after predilation with a balloon catheter. Dissection on OCT appears as the presence of an intimal flap. A thin, well-demarcated linear separation of the intima is observed (marked with *). (**B**) Image of a high-risk plaque. MLA < 3.5 mm^2^, TCFA with fibrous cap thickness < 75 μm, lipid arc > 180° (red semicircle), and macrophage infiltration (pink circle). (**C**) Image of ISR (after dilatation) with features of neoatherosclerosis. A thick, heterogeneous neointimal tissue is observed (blue arrowheads), replacing the typical homogeneous pattern of early hyperplasia. Green arrows highlight the stent struts. (**D**) Layered plaque: a heterogeneous layer (yellow +) overlying a lipid plaque, indicating healing of a recent plaque rupture.

**Figure 3:**
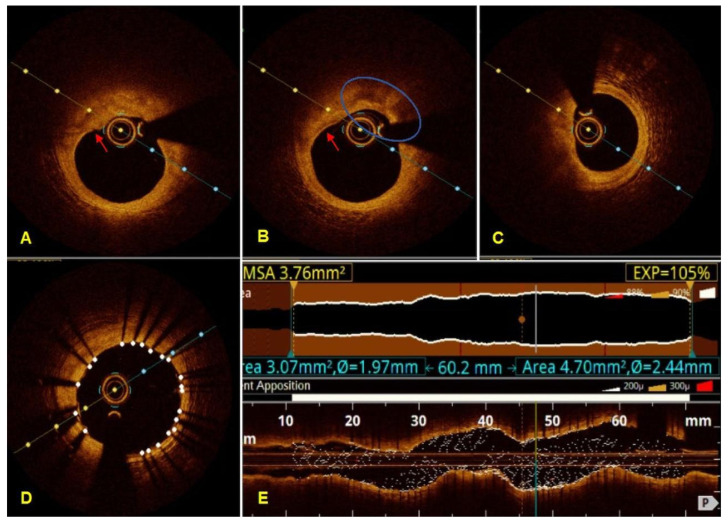
Optical coherence tomography of the LAD in a patient with MINOCA. Cross-sectional pre-PCI images: (**A**) thin-cap atheroma with an irregular luminal surface, suggestive of endothelial denudation (red arrow); (**B**) thin-cap atheroma (red arrow) with abundant macrophages (blue circle); (**C**) plaque with a lipid arc greater than 180°; (**D**) post-PCI cross-sectional image at the site of minimal lumen area, showing an MLA of 7.41 mm^2^; (**E**) Post-PCI OCT image with luminal profile and stent rendered in longitudinal OCT image using cone reference mode, showing the smallest expansion point at 105% of the target expansion (numbers in yellow). The OCT luminal profile indicates the required total stent length of 60.2 mm (numbers in blue). LAD—left anterior descending ar-tery; PCI—percutaneous coronary intervention; MLA—minimum luminal area; OCT—optical coherence tomog-raphy.

Beyond its role in procedural guidance, OCT provides detailed morphological information that supports mechanistic understanding of ACS presentations (Figure 2). OCT can identify plaque rupture, plaque erosion, spontaneous coronary artery dissection, intracoronary thrombus, and stent-related failure mechanisms, including underexpansion, neointimal hyperplasia, strut fracture, and tissue protrusion [35,36,37,38,39,40]. This diagnostic capability is particularly valuable in selected clinical scenarios, such as ACS with angiographically ambiguous lesions, suspected stent failure, myocardial infarction with non-obstructive coronary arteries (MINOCA) (Figure 3), or spontaneous coronary artery dissection.

In the diagnosis of thrombosis and the personalization of antithrombotic therapy, OCT enables precise identification of thrombus, plaque rupture, or erosion, neointima, and the underlying mechanisms of acute coronary syndrome [31,36,37,38]. This morphological characterization is essential for guiding the selection and duration of antiplatelet or antithrombotic therapy, particularly in patients at high risk for ischemic or bleeding events [31,38]. OCT also optimizes stent implantation by reducing thrombosis and ischemic com- plications through ensuring adequate stent expansion and apposition and by detecting issues such as dissection or tissue protrusion [37,38].

However, it is important to emphasize that the identification of plaque morphology or thrombotic features by OCT does not, by itself, mandate specific therapeutic interventions beyond guideline-directed medical therapy and optimized PCI. While OCT-derived findings may inform risk stratification and support individualized clinical reasoning, their direct role in guiding the selection, intensity, or duration of antithrombotic therapy has not been established in outcome-driven randomized trials.

In summary, OCT is a guideline-supported imaging modality for guiding PCI in ACS and complex coronary lesions, with demonstrated benefits in procedural optimization and clinical outcomes when used for this purpose (Class IIa, Level of Evidence A). Its additional value in plaque characterization and mechanistic assessment is well supported by observational and mechanistic studies, but these applications should be interpreted as adjunctive and investigational rather than as indications for definitive therapeutic decision-making.

### 4.4. Comparison of IVUS, OCT, and Angiography: Clinical Outcomes

Randomized clinical trials and contemporary meta-analyses have demonstrated that intracoronary imaging–guided PCI using either IVUS or OCT is associated with improved clinical outcomes compared with conventional angiography-guided PCI, particularly in patients receiving drug-eluting stents for complex coronary lesions and ACS [33,40,41,42,43]. IVUS-guided PCI has shown consistent reductions in target vessel revascularization (odds ratio 0.69; 95% CI 0.54–0.87) and major adverse cardiovascular events, whereas OCT-guided PCI demonstrates similar benefits, including a significant reduction in stent thrombosis (odds ratio 0.49; 95% CI 0.26–0.92) and composite ischemic outcomes in patients with complex lesions. Overall, the available evidence indicates that IVUS and OCT provide comparable benefits with respect to hard clinical endpoints, with no statistically significant differences between the two modalities in mortality or reinfarction across the most recent meta-analyses [33,41,43]. In patients with ACS, both IVUS- and OCT-guided PCI are associated with lower rates of target vessel failure, reinfarction, and composite ischemic events, with procedural safety comparable to angiography-guided PCI and no increase in periprocedural complications [33,42]. Beyond their role in procedural guidance, IVUS and OCT differ substantially in their ability to characterize plaque morphology. OCT offers markedly higher spatial resolution than IVUS, enabling more detailed visualization of superficial plaque features, including lipid-rich plaques, thin-cap fibroatheroma, fibrous cap thickness, plaque erosion, plaque rupture, and intracoronary thrombus, which are frequently implicated in ACS pathophysiology [41,44,45,46,47]. Comparative studies have shown higher sensitivity of OCT for detecting lipid-rich plaques compared with IVUS (e.g., approximately 85% vs. 59%, *p* = 0.03), and OCT remains the only modality capable of directly measuring fibrous cap thickness, a key marker of plaque instability [45,47]. OCT also detects thrombus, plaque erosion, and plaque rupture more frequently and with greater accuracy than IVUS [21,46,47].

Importantly, the identification of lipid-rich plaques or other vulnerability features by OCT primarily informs mechanistic understanding and prognostic risk stratification. At present, these findings do not mandate plaque-directed therapies with proven outcome benefit beyond guideline-directed management of ACS. Once high clinical risk is established, interventions with demonstrated effectiveness include timely revascularization when indicated, procedural optimization during PCI (including imaging-guided stent sizing and expansion in complex lesions), and comprehensive secondary prevention with evidence-based pharmacotherapy, such as antiplatelet therapy tailored to ischemic and bleeding risk, intensive lipid-lowering therapy, and aggressive modification of cardiovascular risk factors. Prospective outcome-driven trials are still needed to determine whether OCT-defined vulnerability features should directly guide additional therapeutic intensification beyond current guideline-based strategies.

In contrast, IVUS remains particularly valuable for assessing overall plaque burden, vascular remodeling, and deeper plaque components, and it is widely used for procedural optimization during PCI. However, IVUS is less sensitive for detailed characterization of superficial lipid composition within the plaque [26,42,48]. The main limitations of OCT include its lower tissue penetration depth and the requirement for contrast injection, which may restrict its use in certain coronary anatomies or in patients with renal dysfunction [26]. Although OCT is superior to IVUS for detailed assessment of plaque morphology and vulnerability features, plaque characterization by OCT primarily informs risk stratification and mechanistic understanding rather than outcome-driven treatment decisions [41,47].

### 4.5. Integration of Artificial Intelligence into Optical Coherence Tomography

Clinical studies applying real-time artificial intelligence (AI) algorithms to the analysis of intracoronary optical coherence tomography (OCT) images have shown that AI improves the identification of high-risk plaques and enhances prognostic stratification. However, robust evidence of additional clinical benefits—such as reductions in ischemic or bleeding events—compared with conventional operator-guided OCT is still lacking. The PECTUS-AI study demonstrated that AI-automated OCT analysis identifies thin-cap fibroatheromas (TCFAs) with greater prognostic accuracy than manual interpretation, and that these findings are associated with an increased risk of adverse cardiovascular events in patients with myocardial infarction. Automated, whole-segment coronary evaluation using AI showed a high negative predictive value for adverse events, suggesting utility in risk stratification and potential applicability for guiding individualized therapies [49].

**Figure 4:**
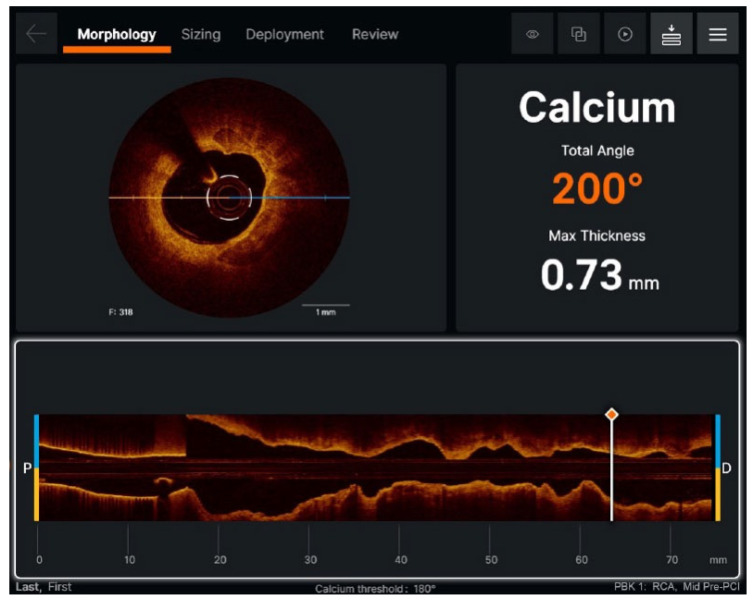
Ultreon™ 2.0 software (Abbott). Imaging platform using artificial intelligence to provide automated assessment of multiple parameters.

AutoOCT, validated across multiple cohorts, has demonstrated equivalence to expert analysis in identifying plaque characteristics and assessing statin response, in addition to detecting morphological parameters associated with elevated risk, such as minimal luminal area, lipid arc, and fibrous cap thickness [50]. However, these studies are observational or focused on technical validation, with no direct comparison of clinical outcomes between AI-guided strategies and those guided by human operators. In the field of AI-enhanced OCT, the Ultreon platform is also notable. Ultreon is a software system used for OCT image analysis that assists in interpreting imaging data by enabling automated assessment of parameters such as luminal area, calcification extent, and stent optimization. It is used to improve the evaluation and optimization of image-guided coronary interventions (Figure 4). According to the American Heart Association, the adoption of AI in cardiovascular imaging requires validation through randomized clinical trials to demonstrate benefits in clinically relevant outcomes, such as ischemic and bleeding events, before broad recommendations can be made [51]. Therefore, there is no definitive clinical evidence that real-time AI–assisted OCT analysis reduces ischemic or bleeding events compared with conventional operator-guided use in patients with acute coronary syndromes and complex comorbidities.

## 5. Integration of Artificial Intelligence (AI) into Clinical Practice with a Focus on Thrombosis Prevention

The integration of artificial intelligence (AI) into clinical practice is rapidly evolving, driven by the exponential growth of clinical, laboratory, genetic, and imaging data in cardiovascular medicine and hematology. AI has the potential to enhance diagnostic accuracy, risk stratification, and clinical decision support in thrombosis; however, its implementation in routine clinical practice requires rigorous validation, robust governance structures, and standardized frameworks to ensure safety, effectiveness, and equity [52,53,54,55,56].

In diagnosis and risk stratification, artificial intelligence enables integrated analysis across multiple clinical, laboratory, genetic, biomarker, and imaging data domains. In observational and retrospective studies, machine-learning models applied to these data—including optical coherence tomography and computed tomography angiography—have demonstrated moderate improvements in predictive performance, with reported area under the curve values typically ranging from approximately 0.75 to 0.85 for the prediction of thrombotic or adverse cardiovascular events [54,57,58,59]. Imaging-based AI models have shown sensitivities of approximately 80–90% for identifying high-risk plaque features, although specificity has varied across studies. While AI-based risk models, biosensors, and digital twin approaches have demonstrated feasibility and modest improvements in discrimination and risk reclassification compared with conventional clinical scores, these findings remain derived primarily from retrospective analyses, and prospective outcome-driven randomized trials are still lacking [58,60].

AI-based predictive models, advanced biosensors, and digital twin approaches have shown feasibility in integrating clinical, genetic, and environmental data to generate personalized thrombotic risk profiles. While these tools illustrate the potential of AI-driven personalization, their clinical utility remains investigational, as prospective evidence demonstrating incremental benefit over established risk stratification strategies is currently limited [52,61]. With respect to therapeutic decision support, AI has been explored as an adjunctive tool to assist clinicians in risk estimation and treatment planning. However, these applications should not be interpreted as substitutes for guideline-directed clinical judgment, as they have not yet been validated in randomized trials demonstrating improvements in hard clinical outcomes [52,55,56].

Several important limitations currently constrain the clinical adoption of AI in thrombosis management, including the lack of outcome-driven randomized trials, algorithmic bias related to non-representative training datasets, regulatory and ethical challenges, and barriers to seamless integration into real-world clinical workflows. Consequently, the role of AI in therapeutic decision-making should be considered evolving and investigational, pending confirmation in prospective studies and validation across diverse patient populations [52,53,56].

## 6. Emerging Pharmacological Strategies: Factor XI-Targeted Anticoagulants

Emerging pharmacological strategies targeting factor XI aim to dissociate antithrombotic efficacy from bleeding risk, based on the biological rationale that factor XI plays a more prominent role in thrombus propagation than in physiological hemostasis. Factor XI (FXI/FXIa) inhibitors therefore represent a promising investigational approach for thrombosis prevention, particularly in patients at high bleeding risk or with contraindications to conventional anticoagulants [62,63,64]. Several FXI-targeted agents are currently under clinical development, including small oral molecules (asundexian, milvexian), monoclonal antibodies (abelacimab, osocimab), and antisense oligonucleotides (fesomersen). In phase II and selected phase III trials, as well as in meta-analyses, FXI inhibitors have demonstrated favorable safety profiles, with significant reductions in bleeding compared with enoxaparin and, in some settings, a trend toward lower bleeding rates compared with direct oral anticoagulants (DOACs), without a clear excess of thromboembolic events [65,66]. However, these studies were not primarily designed or powered to establish superiority for ischemic outcomes across broad cardiovascular populations, as shown in Table 3.

In high-risk clinical settings such as orthopedic surgery, end-stage renal disease, atrial fibrillation, and selected acute coronary syndrome cohorts, FXI inhibitors have shown efficacy that appears comparable to conventional anticoagulation, accompanied by lower bleeding rates, particularly among patients with elevated hemorrhagic risk or multiple comorbidities [63,67,68]. Nevertheless, the heterogeneity of studied populations and clinical contexts limits direct extrapolation of these findings to routine cardiovascular practice.

In acute coronary syndromes and high cardiovascular risk settings, agents such as asundexian, milvexian, and abelacimab have demonstrated, in meta-analyses and multicenter studies, a reduction in bleeding risk compared with enoxaparin (relative risk for bleeding 0.42) and no significant increase in thromboembolic events (relative risk 0.59), as well as a trend toward reduced bleeding compared with DOACs [65,66]. Importantly, these observations derive largely from phase II studies, pooled analyses, and exploratory endpoints, rather than from definitive outcome-driven trials.

Among patients with multiple comorbidities, including renal dysfunction, cancer, or atrial fibrillation, FXI inhibitors have maintained a favorable safety profile, with low rates of major bleeding and efficacy broadly comparable to standard anticoagulant therapy [65,66,69]. Available data also suggest that, in the context of concomitant antiplatelet therapy, FXI inhibitors do not substantially increase major bleeding risk when compared with standard anticoagulation, even in patients receiving dual antiplatelet therapy after acute coronary syndrome [62,70]. However, these findings should be interpreted cautiously, given the limited duration of follow-up and the absence of large-scale randomized outcome trials in this population.

Crucially, FXI inhibitors remain investigational for most cardiovascular indications, including acute coronary syndromes. Their definitive role in ACS management, secondary prevention of myocardial infarction, and ischemic stroke prevention awaits the completion of large, adequately powered phase III randomized clinical trials that are currently ongoing [71,72]. These studies are expected to clarify not only efficacy and safety, but also optimal patient selection, interaction with antiplatelet therapy, and long-term clinical outcomes [62,71]. In summary, FXI inhibitors represent a promising investigational strategy with the potential to reduce bleeding while preserving antithrombotic efficacy in selected high-risk populations. Despite encouraging phase II and early phase III data, these agents have not yet been incorporated into guideline-directed cardiovascular care, and their definitive positioning in acute coronary syndromes and other cardiovascular settings will depend on the results of ongoing large-scale randomized trials.

## 7. Personalized Therapeutic Approaches and Future Directions in the Integration of Diagnosis and Therapy

In antithrombotic therapy, significant advances have been achieved with the use of potent P2Y12 inhibitors, aspirin de-escalation strategies, and the introduction of direct oral anticoagulants, which offer a more favorable safety profile in patients with complex comorbidities such as renal insufficiency or cancer [4,5,73]. Contemporary guidelines, including those from the European Society of Cardiology, recommend shortening the duration of triple antithrombotic therapy after acute coronary syndrome in patients with atrial fibrillation, prioritizing DOACs and P2Y12 inhibitors to minimize bleeding risk [73]. Personalized treatment strategies increasingly rely on established clinical tools, including the DAPT score, platelet function testing, and selected genetic markers, to balance ischemic and bleeding risks in individual patients [6,8,9]. In this context, intracoronary imaging—particularly optical coherence tomography—has substantially improved the mechanistic understanding of acute coronary syndromes by enabling detailed characterization of plaque morphology, thrombus, plaque rupture or erosion, and post-stent complications, thereby supporting individualized procedural and medical decision-making [31,36,37,39,74]. Advances in biomarker research, including plasma proteomics and multimarker panels, have further refined thrombotic and bleeding risk stratification, allowing more accurate identification of patients who may benefit from intensified or emerging antithrombotic strategies [1,75]. These approaches complement, rather than replace, established guideline-directed therapies and clinical risk scores.

Artificial intelligence–based tools may, in the future, facilitate integration of clinical, imaging, and biomarker data to support personalized therapeutic strategies. However, as discussed in Section 4, the application of AI in this context remains investigational and has not yet been shown to improve clinical outcomes in randomized trials. Accordingly, current personalized antithrombotic strategies should continue to be grounded in validated clinical tools, imaging modalities, and guideline-supported pharmacological therapies.

Emerging pharmacological strategies targeting factor XI represent a promising investigational approach aimed at dissociating antithrombotic efficacy from bleeding risk. In phase II and selected phase III clinical trials and meta-analyses, FXI inhibitors have demonstrated significant reductions in bleeding compared with enoxaparin, with reported risk ratios for major or clinically relevant non-major bleeding of approximately 0.40–0.50, while maintaining comparable protection against thromboembolic events (risk ratio for thromboembolism approximately 0.55–0.65).

Similarly, pooled analyses of published and ongoing phase III studies suggest that FXI inhibitors provide efficacy that is comparable to, or in some settings numerically greater than, conventional anticoagulation with enoxaparin or direct oral anticoagulants for the prevention of thromboembolic events, while achieving a consistent and clinically meaningful reduction in bleeding risk. Across these studies, bleeding risk reductions on the order of 50–60% have been reported (risk ratio approximately 0.40–0.50), particularly in patients with multiple comorbidities and in those receiving concomitant antiplatelet therapy, without a statistically significant increase in thromboembolic events [52,61]. However, despite these favorable safety signals, FXI inhibitors remain investigational for most cardiovascular indications. Their definitive role in acute coronary syndromes and other high-risk cardiovascular settings awaits confirmation in large, adequately powered outcome-driven randomized clinical trials designed to establish net clinical benefit and optimal patient selection.

## 8. Conclusions

Advances in biomarkers, intracoronary imaging, artificial intelligence, and emerging antithrombotic therapies have substantially expanded the understanding of thrombosis in acute coronary syndromes and in patients at high cardiovascular risk. High-sensitivity cardiac troponins and natriuretic peptides remain the most robust and guideline-supported biomarkers for diagnosis and prognostic stratification, whereas inflammatory markers and multimarker models provide incremental prognostic information but still lack definitive validation for routine therapeutic guidance.

Intracoronary imaging techniques, particularly intravascular ultrasound and optical coherence tomography, play an established role in guiding percutaneous coronary intervention in selected patients, leading to improved procedural optimization and clinical outcomes when compared with angiography-guided strategies. Beyond procedural guidance, advanced plaque characterization—especially with OCT—enhances mechanistic understanding and risk stratification but does not yet support plaque-directed therapeutic interventions. Near-infrared spectroscopy further complements risk assessment by identifying lipid-rich plaques, although its prognostic value is derived predominantly from observational evidence and awaits confirmation in randomized outcome-driven studies.

Artificial intelligence represents a rapidly evolving tool for integrating clinical, laboratory, and imaging data, with encouraging results in retrospective and observational analyses. However, its role in thrombosis management remains investigational, as prospective randomized trials demonstrating improvements in clinically meaningful outcomes are currently lacking.

In the therapeutic domain, factor XI inhibitors constitute a promising investigational strategy aimed at reducing bleeding while preserving antithrombotic efficacy. Despite favorable safety signals and encouraging phase II and early phase III data, their definitive role in acute coronary syndromes and other cardiovascular settings awaits validation in large, outcome-driven randomized clinical trials.

Overall, the convergence of biomarkers, intracoronary imaging, and emerging analytical tools highlights the potential for more precise and individualized cardiovascular care. Nevertheless, careful interpretation of existing evidence, rigorous validation, and alignment with guideline-directed practice remain essential to ensure safe and effective integration of these approaches into routine clinical care.

## 9. Future Directions

Despite substantial advances, several key questions remain unanswered. Further studies are needed to define which patient subgroups derive the greatest clinical benefit from advanced intracoronary imaging, emerging antithrombotic strategies such as factor XI inhibition, and integrated biomarker approaches. Large, outcome-driven randomized trials will be essential to clarify whether plaque vulnerability features, multimarker profiles, or artificial intelligence–based risk models can meaningfully guide therapeutic intensification beyond current guideline-directed care. In parallel, future research should focus on the integration of multi-omic data into clinically actionable frameworks and on addressing practical challenges related to implementation, including workflow integration, interpretability, and equitable access. Addressing these gaps will be critical for translating technological and biological advances into tangible improvements in patient outcomes.

## Figures and Tables

**Table 1:** Biomarkers in Acute Coronary Syndromes and High-Risk Cardiovascular Scenarios.

Biomarker	Main Clinical Application	Prognostic Value	Role in Therapeutic Personalization	Level of Evidence/Guidelines
hs-cTn	Diagnosis of ACS; risk stratification	High	Guides invasive strategy and intensity of antithrombotic therapy	High—recommended by ACC/AHA/ESC guidelines
BNP/NT-proBNP	Heart failure assessment and global risk	High	Identifies patients at higher risk and worse prognosis	High—widely validated
Homocysteine	Assessment of residual thrombotic risk	Moderate	Potential role in multimarker models	Moderate—validation ongoing
hsCRP	Vascular inflammation and plaque instability	Moderate	May support decisions on therapy intensification	Moderate
IL-6	Systemic inflammation and vulnerable plaques	Moderate	Promising for inflammatory risk stratification	Moderate
GDF-15	Thrombotic and bleeding risk	Moderate–High	Supports ischemic–bleeding risk balance	Moderate
sCD40L	Platelet activation	Low–Moderate	Complementary biomarker	Low
Multimarker models	Integrated risk stratification	High (in studies)	Foundation for personalized medicine	Emerging
Omics biomarkers (proteomics, genomics)	Molecular diagnosis and risk prediction	High potential	Advanced therapeutic personalization	Translational research

Legend: hs-cTn: high-sensitivity cardiac troponin; ACS: acute coronary syndrome; ACC: American College of Cardiology; AHA: American Heart Association; ESC: European Society of Cardiology; BNP: B-type natriuretic peptide; NT-proBNP: N-terminal pro–B-type natriuretic peptide; hsCRP: high-sensitivity C-reactive protein; IL-6: Interleukin-6; GDF-15: growth differentiation factor-15; sCD40L = soluble CD40 ligand.

**Table 2:** Intracoronary Imaging Modalities in Acute Coronary Syndromes and High-Risk Patients.

Modality	Main Advantages	Limitations	Key Clinical Applications	Clinical Evidence
Coronary angiography	Widely available; rapid	Two-dimensional lumen view; poor plaque characterization	Initial diagnosis and basic PCI guidance	Historical standard
Intravascular ultrasound (IVUS)	Assesses total plaque burden and vascular remodeling	Limited resolution for plaque composition	Stent optimization; complex lesions	High
Optical coherence tomography (OCT)	Very high resolution; detects TCFA, thrombus, erosion, rupture	Limited penetration depth; requires contrast	Vulnerable plaque characterization; ACS; MINOCA	High
NIRS-IVUS	Identifies lipid-rich plaques	Evidence mainly observational	Future risk stratification	Moderate
AI-assisted OCT	Automated analysis; improved reproducibility	No proven impact on clinical outcomes	High-risk plaque identification	Emerging
Coronary CT angiography	Non-invasive anatomical assessment	Lower intracoronary resolution	Initial anatomical evaluation	Moderate
Cardiac magnetic resonance	Functional and tissue characterization	Limited for intracoronary thrombus	Etiological differentiation of ACS	Moderate

**Table 3:** Factor XI (FXI/FXIa) Inhibitors: Characteristics and Clinical Evidence.

Drug	Class	Route of Administration	Main Studied Scenarios	Bleeding Profile	Clinical Development Stage
Asundexian	Small-molecule FXIa inhibitor	Oral	AF, ACS, secondary prevention	Reduced vs. DOACs	Phase III
Milvexian	Small-molecule FXIa inhibitor	Oral	Venous thromboembolism, AF	Reduced	Phase III
Abelacimab	Monoclonal antibody	Subcutaneous/IV	AF, high-risk patients	Very low	Phase III
Osocimab	Monoclonal antibody	IV	Orthopedic surgery	Reduced	Phase II–III
Fesomersen	Antisense oligonucleotide	Subcutaneous	Thrombosis prevention	Reduced	Phase II
Comparison with DOACs	—	—	AF, ACS, VTE	Lower bleeding risk	Emerging evidence

## Data Availability

No new data were created or analyzed in this study. Data sharing is not applicable to this article.

## Abbreviations

The following abbreviations are used in this manuscript.

ACSs	acute coronary syndromes
AI	artificial intelligence
DAPT	dual antiplatelet therapy
DOACs	direct oral anticoagulants
IVUS	intravascular ultrasound
LAD	left anterior descending
MINOCA	myocardial infarction with non-obstructive coronary arteries
MLA	minimum lumen area
NIRS	Near-infrared spectroscopy
NPs	Natriuretic peptides
OCT	optical coherence tomography
PCI	percutaneous coronary intervention
TCFAs	thin-cap fibroatheromas
